# Randomized Controlled Trials Conducted in Japan as a Comparison with Top-ranking Countries

**DOI:** 10.2188/jea.11.46

**Published:** 2007-11-30

**Authors:** Mahbubur Rahman, Miho Sekimoto, Takeshi Morimoto, Tsuguya Fukui

Since its introduction in 1948, the randomized controlled trial (RCT) is now widely accepted as a vehicle for providing evidence of the highest level together with the least biased results regarding the efficacy of therapeutic or preventive interventions. In fact, it constitutes the main pillar of evidence-based medicine. A large number of participants and double-blinding are the two important criteria which have significantly raised precision and eliminated bias, respectively. However, RCTs conducted in Japan are allegedly said to be characterized by fewer participants and to be less likely double-blinded. Our present investigation aimed 1) to identify the 10 top-ranking countries which have produced most of the RCT results and 2) to compare RCTs conducted in Japan with those conducted in other top-ranking countries in terms of the number of participants and the proportion of double-blind trials.

**Figure 1.**
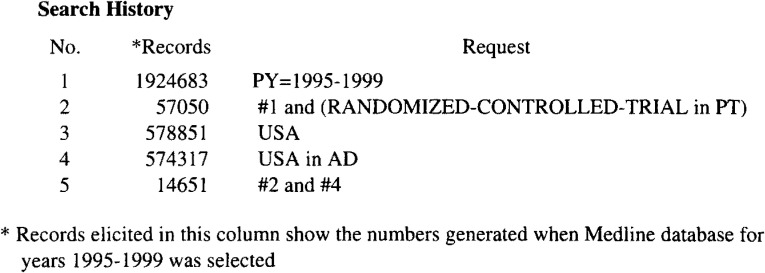
Search history for the retrieval of RCTs conducted in the USA. A similar procedure was used for all countries to obtain the top-ranking 10 countries.

The Medline database for 1995-1999 was searched in September 2000 using WINSPIRS. We selected 1995 as the first year, because the data on RCT, available through WINSPIRS were incomplete before that year. For example, according to the search results, the average number of RCTs conducted in the USA during 1990-1994 was only 26 per year while it was 3,033 per year during 1995-1998. Again, before 1990 reliable data on authors’ affiliations were not available through WINSPIRS, so that we could only include these data published after 1994. Search formula were illustrated in Figure 1. The same commands were used to determine the total (“raw”) number of RCTs conducted by each country for selection of the top 10 countries which conducted most of the RCTs. After that, from among the total RCTs obtained through a snap search of the Medline database, a random sample of 100 was taken for each of the 10 countries. Next, hard copies of the abstracts were obtained to extract data for the following variables: whether it was a true RCT, language of the article, blinding method (single, double or no blinding), and the number of participants. The percentage of true RCTs thus obtained was then used to calculate adjusted (“true”) numbers of RCTs.

Average raw numbers as well as adjusted (“true”) numbers of RCTs published per year in the world’s 10 top-ranking countries are shown in Table 1. The proportions of “true” RCTs ranged from 54 to 77%, and those for double-blinded RCTs ranged from 16 to 36%. The number of participants per RCT in Japan was 89, and the proportion for double-blinding was 16%. In this regard, Japan lags behind all the top-ranking 9 countries other than Australia. The calculation of the “true” number of RCTs showed that the USA accounted for almost half of the total “true” RCTs published from the 10 countries while Japan contributed only 5%. All the RCTs published from the top-ranking 10 countries were in English except five from France and two from Japan, which were published in French and Japanese, respectively.

**Table 1.** RCTs conducted in the world’s 10 top-ranking countries.

Country	Average “raw” number of RCTs per year	Confirmed by reading 100 randomly selected abstracts for each country			Average “true” number of RCTs per year (percentage contribution among 10 countries)	

		Proportion of true RCTs (%)	Proportion of double-blind RCTs (%)	Average number of participants		
USA	2930	63	26	267	1846	(45%)
UK	533	77	33	127	410	(10%)
Germany	464	68	35	263	316	(8%)
Italy	425	73	22	132	310	(8%)
Canada	364	67	21	215	244	(6%)
The Netherlands	359	66	22	133	237	(6%)
Fance	314	61	30	137	192	(5%)
Sweden	305	74	36	155	226	(6%)
Japan	276	71	16	89	196	(5%)
Australia	196	54	17	96	106	(3%)

The results could be different if normalized to population, gross domestic product, and physician population, as reported in some studies conducted on biomedical publications^1^^-^^5^^)^. In this regard, the current data is only on the actual numbers of RCTs, showing an overall contribution of each country.

The results show the number of RCTs only, irrespective of their quality or usefulness. Some RCTs may have been published more than once either in the same or different languages; so there might be overestimation of the number of RCTs for all countries, not any particular one. Again, many RCTs are multinational trials and only corresponding authors’ affiliations were taken into account, so that the number of RCTs might be higher for some countries which have more resources for organizing multinational trials. At any rate, it would be helpful to have a general impression about the countries producing most of the RCTs.

It is undeniable that Japanese RCTs contain fewer participants and are prone to be unmasked compared with those of other developed countries. Further studies on the causes behind these shortcomings are clearly warranted.
